# Perspectives of Inpatients With Cirrhosis and Caregivers on Using Health Information Technology: Cross-sectional Multicenter Study

**DOI:** 10.2196/24639

**Published:** 2021-04-09

**Authors:** Chathur Acharya, Tejasav S Sehrawat, Deborah B McGuire, Jawaid Shaw, Andrew Fagan, Sara McGeorge, Amy Olofson, Melanie B White, Edith Gavis, Patrick S Kamath, Lori Bergstrom, Jasmohan Singh Bajaj

**Affiliations:** 1 Division of Gastroenterology, Hepatology and Nutrition Virginia Commonwealth University Richmond, VA United States; 2 McGuire VA Medical Center Richmond, VA United States; 3 Division of Gastroenterology and Hepatology Mayo Clinic School of Medicine Rochester, MN United States

**Keywords:** hepatic encephalopathy, cirrhosis, outcomes, acceptance, PatientBuddy, ascites, readmissions, hepatic, encephalopathy

## Abstract

**Background:**

Health information technology (IT) interventions to decrease readmissions for cirrhosis may be limited by patient-associated factors.

**Objective:**

The aim of this study was to determine perspectives regarding adoption versus refusal of health IT interventions among patient-caregiver dyads.

**Methods:**

Inpatients with cirrhosis and their caregivers were approached to participate in a randomized health IT intervention trial requiring daily contact with research teams via the Patient Buddy app. This app focuses on ascites, medications, and hepatic encephalopathy over 30 days. Regression analyses for characteristics associated with acceptance were performed. For those who declined, a semistructured interview was performed with themes focused on caregivers, protocol, transport/logistics, technology demands, and privacy.

**Results:**

A total of 349 patient-caregiver dyads were approached (191 from Virginia Commonwealth University, 56 from Richmond Veterans Affairs Medical Center, and 102 from Mayo Clinic), 87 of which (25%) agreed to participate. On regression, dyads agreeing included a male patient (odds ratio [OR] 2.08, *P*=.01), gastrointestinal bleeding (OR 2.3, *P*=.006), or hepatic encephalopathy admission (OR 2.0, *P*=.01), whereas opioid use (OR 0.46, *P*=.03) and alcohol-related etiology (OR 0.54, *P*=.02) were associated with refusal. Race, study site, and other admission reasons did not contribute to refusing participation. Among the 262 dyads who declined randomization, caregiver reluctance (43%), perceived burden (31%), technology-related issues (14%), transportation/logistics (10%), and others (4%), but not privacy, were highlighted as major concerns.

**Conclusions:**

Patients with cirrhosis admitted with hepatic encephalopathy and gastrointestinal bleeding without opioid use or alcohol-related etiologies were more likely to participate in a health IT intervention focused on preventing readmissions. Caregiver and study burden but not privacy were major reasons to decline participation. Reducing perceived patient-caregiver burden and improving communication may improve participation.

**Trial Registration:**

ClinicalTrials.gov NCT03564626; https://www.clinicaltrials.gov/ct2/show/NCT03564626

## Introduction

Patients with cirrhosis often require expensive inpatient and outpatient care, mainly centered around complications related to hepatic encephalopathy, variceal bleeding, ascites, and medication management [1]. A critical component of optimal care is the education of patients and their caregivers, and communication with the clinical team [2]. Educational strategies for and involvement of caregivers in care processes are often neglected in favor of dispensing medications and scheduling appointments at the time of discharge [3,4]. Tools that could enhance delivery of care and maintain a link between health care providers and patients/caregivers are needed to improve clinical management and outcomes. In a prior single-center study, we evaluated the acceptability and performance of the Patient Buddy app for preventing readmissions in patients with cirrhosis [5]. The app involves two components recorded by patients and their caregivers on EncephalApp, a separate app designed to assess cognitive function in patients with cirrhosis: (1) daily postdischarge smartphone entry for 30 days of weight, abdominal girth, temperature, and medication adherence; and (2) weekly results of responses to questions related to orientation in time, place, and person [6,7]. The data are transmitted via a Health Insurance Portability and Accountability Act (HIPAA)-compliant server to central iPads, which the study team then review for assessment of alarm values that are used for communication with patients and caregivers and to determine if action is required. The central iPads are reviewed multiple times a day. In our single-center study, we found that use of the app alerted health care providers to early evolution of hepatic encephalopathy, and was largely favorably received by the patients and caregivers using the app [5].

We are currently performing a multicenter randomized trial of the Patient Buddy app at three clinical sites in patients with cirrhosis and their caregivers. The purpose of this study was to examine the perspectives of both patients and caregivers when considering use of this intensive daily health information technology (IT). Their willingness to use the app has direct bearing on patient participation in this and other multisite trials, and ultimately on the health care team’s ability to monitor clinical status, reduce readmissions, and improve outcomes in cirrhosis.

## Methods

We designed this study to assess the reasons for potential refusal to better inform recruitment approaches. We performed semistructured interviews in patient-caregiver dyads in the ongoing multicenter randomized study of cirrhosis inpatients and their caregivers on use of the Patient Buddy app to prevent 30-day readmissions. The randomized study is being performed in tertiary-care hospitals serving three different populations: veterans at Veterans Affairs (VA) Medical Center (Richmond, VA), uninsured/underinsured patients at a state safety net institution (Virginia Commonwealth University [VCU], Richmond, VA), and insured patients receiving care at a private group practice (Mayo Clinic, Rochester, MN). Regardless of the setting, the standard of care for all three hospitals requires setting up a postdischarge follow-up appointment within 1 month and a phone call within 1 week.

We screened for inpatients who met the following eligibility criteria: (1) diagnosis of cirrhosis as defined by liver biopsy, overt hepatic decompensation (hepatic encephalopathy, ascites, variceal bleeding, or jaundice), or radiological features of cirrhosis and endoscopic features of varices in patients with chronic liver disease; and (2) those who were admitted nonelectively and had adult cohabitating caregivers. Patients excluded for screening were those with an active alcohol use disorder, without caregivers or a stable home, on hemodialysis, discharged to a facility or hospice, inadequate internet connection at home, or an unclear diagnosis of cirrhosis. For patients who met the eligibility criteria, a member of the study team approached the patient and caregiver in person or via telephone while the patient was still hospitalized and explained the study in detail. This explanation included a discussion with the patient-caregiver dyad that emphasized the necessity of being in contact with the study team after discharge, and the potential educational value of the app and its implications for communication with the team on clinical issues. Another aspect of the discussion involved a demonstration of the app and the need for data entry of critical medications (ie, hepatic encephalopathy medications, antibiotics, beta blockers, diuretics, and other cirrhosis-related medications), weight and abdominal girth, and orientation with EncephalApp.

The intervention consisted of the Patient Buddy app coupled with EncephalApp. For the dyad to participate in the multisite randomized trial postdischarge, they had to agree to be trained individually while the patient was hospitalized, including instructions for data entry in the app; measuring body weight, temperature, and girth (using scales, measuring tapes, and thermometers provided by the study team, respectively); assessing orientation; and training to caregivers in administration of EncephalApp. If the dyad consented to participate, they were randomly assigned to one of three groups (standard of care, health IT intervention with as-needed follow-up, or health IT intervention with scheduled outpatient visits and calls within 30 days of discharge). If assigned to either of the health IT groups, the app was activated upon discharge on a smartphone provided as part of the study.

In the randomized study, we collect data from all dyads who are approached for participation, including demographic and clinical characteristics, reasons for admission, and current patient medications. Dyads indicating that they did not want to be randomized were then asked if they would participate in a semistructured qualitative interview that was designed and supervised by an expert (DM) to explore reasons for deciding not to participate and to help ascertain issues related to use of health IT as opposed to research as a whole that could guide future similar studies.

The patients and caregivers were asked about their reasons for unwillingness divided into five categories (demands related to technology, caregiver reluctance, perceived burden of the study, transport and logistics, privacy-related, or other) and other reasons through the interview questions prompted by the coordinators with qualitative responses allowed. All potential inpatients were approached in person. The study coordinators carried out the interview, and every effort was made to perform these analyses within 1 day for both potential caregivers and patients. The responses were transcribed on an interview form by the study coordinator explaining the study, which also had space for expressing other potential reasons that informed their decision not to participate further. The other reasons included refusal to participate in studies of any kind regardless of the health IT intervention, unspecified, or unwillingness to elaborate further.

In the analysis, we compared the demographic and clinical variables such as cirrhosis characteristics, especially hepatic encephalopathy, current medications, and reasons for admission, in dyads who agreed to participate with those of dyads who did not agree to participate using unpaired *t* tests or χ^2^ tests as appropriate. Finally, a logistic regression analysis was performed with agreement to participate in the randomization process as the dependent variable and disease characteristics as independent variables.

## Results

A total of 349 patient-caregiver dyads were approached at the three sites (191 at VCU, 56 at VA, and 102 at Mayo Clinic), 87 of which (24.9%) agreed to participate. Table 1 shows the characteristics of these dyads and indicates the significantly different variables between those who agreed and declined participation.

**Table 1:** Demographic and clinical characteristics of patients who agreed and did not agree to randomization for the health information technology intervention study.

Characteristics		Agreed to randomization (n=87)	Did not agree to randomization (n=262)	*P* value
Age (years), mean (SD)		58.8 (11.3)	59.2 (11.4)	.78
**Race, n (%)**				.06
	Caucasian	64 (74)	207 (79.0)	
	African American	15 (17)	47 (17.9)	
	Other	8 (9)	8 (3.1)	
Men, n (%)		62 (71)	150 (57.3)	.02
**Comorbidity, n (%)**				.03
	HCV^a^	5 (6)	28 (10.7)	
	HCV+alcohol-related	26 (30)	117 (44.7)	
	Alcohol-related	9 (10)	15 (5.7)	
	NAFLD^b^	25 (29)	60 (22.9)	
	Other	22 (25)	42 (16.0)	
Prior HE^c^, n (%)		56 (64)	151 (57.6)	.27
Lactulose use, n (%)		50 (57)	152 (58.0)	.93
Rifaximin use, n (%)		40 (46)	101 (38.5)	.22
Taking psychoactive medications, n (%)		12 (14)	57 (21.8)	.09
Taking opioids, n (%)		11 (13)	71 (27.1)	.004
MELD^d^ score on admission, mean (SD)		19.5 (6.9)	18.4 (8.4)	.24
**Reason for admission, n (%)**				
	Infection	29 (33)	81 (30.9)	.68
	HE	38 (44)	82 (31.3)	.03
	Renal or metabolic disease	29 (33)	97 (37.0)	.58
	Gastrointestinal bleeding	28 (32)	50 (19.1)	.01
	Other	21 (24)	144 (55.0)	<.001

^a^HCV: hepatitis C virus.

^b^NAFLD: nonalcoholic fatty liver disease.

^c^HE: hepatic encephalopathy.

^d^MELD: Model for End-stage Liver Disease.

Of these 87 dyads, 40 were from VCU, 22 from VA, and 25 from Mayo Clinic. The 262 dyads (75.1%) who decided not to move forward to the randomization process all agreed to participate in the semistructured interview. Of this group, 151 were from VCU, 34 from VA, and 77 from Mayo Clinic. Results from the semistructured interview revealed that 206 dyads had only one reason for not participating, 50 had two reasons, and 6 had three or more reasons. Of these, the majority of the dyads (198/262, 75.6%) agreed to having the interviews performed in person, while the remaining 8 (3.1%) had the potential caregiver interviewed on the phone within 1 day of initial in-person contact with the patient. The most common reason was caregiver reluctance (114/262, 43.5%), followed by perceived study burden (n=82, 31.3%), technology-related issues (n=36, 13.7%), transportation and logistics (n=26, 9.9%), and no specific reason (n=11, 4.2%). Importantly, none of the dyads listed privacy as a concern for their refusal.

Logistic regression was performed using dyads that said “yes” to being randomized and therefore were included in the multisite trial. The significant factors associated with dyads saying yes (Table 1) included male gender (odds ratio [OR] 2.08, *P*=.01), admission for gastrointestinal bleeding (OR 2.3, *P*=.006), or hepatic encephalopathy (OR 2.0, *P*=.01), whereas opioids (OR 0.46, *P*=.03) and alcohol-related etiology (OR 0.54, *P*=.02) showed the reverse pattern. Race, site of recruitment, other reasons for admission, whether or not the potential caregiver was a spouse, education, and use of other medications were not significantly associated with saying “yes” to be randomized.

In the 262 dyads who declined to be randomized, we compared characteristics of patients who refused for specific reasons (Tables 2 and 3). We did not compare those who had some part of this interview performed in person versus telephone due to the small number (n=8) of those interviewed solely by telephone. Of the potential caregivers, the majority were wives (n=90, 34.4%), followed by husbands (n=55, 21.0%), children (n=30, 11.5%), significant others (n=8, 3.1%), parents (n=18, 6.9%), siblings (n=16, 6.1%), and other people (n=45, 17.2%). Of the 262 patients who did not want to participate, 116 (44.3%) had an education below or at high school level, 113 (43.1%) patients had completed undergraduate college education, and 50 (19.1%) patients had masters or higher degrees.

For analyses, we combined spouse/significant others (n=153, 58.4%) and those with high school education or below for comparison with the others. Apart from technology-related demands, there were no significant differences in the demographic variables, cirrhosis severity or etiology, medication use, hepatic encephalopathy status, or reason for admission among patients who refused compared to the rest. Dyads who refused for technology-related reasons were more likely to be older, with lower education in the patient, had a higher Model for End-stage Liver Disease (MELD) score on admission, higher frequency of hepatic encephalopathy, and were less likely to be admitted for gastrointestinal bleeding or liver-unrelated (other) reasons compared with those who did not refuse for technology-related reasons (Table 2). Caregiver reluctance was lower when the potential caregiver was a spouse, but the reverse was found for perceived study burden. Very few dyads expressed that they were refusing randomization because they were not interested in research, which was included in the “no reason” category.

**Table 2:** Reasons for refusal to participate in relation to patient characteristics (N=262).

Characteristic			Caregiver reluctance				Perceived study burden				Technology demands		
			Yes (n=114)		No (n=148)		Yes (n=82)		No (n=180)		Yes (n=36)		No (n=226)
Age (years)^a^, mean (SD)			58.8 (11.8)		59.5 (11.1)		58.0 (11.9)		59.7 (11.2)		62.9 (9.3)		58.7 (11.4)
**Race, n (%)**													
	Caucasian	88 (77.2)		89 (60.1)		68 (83)		139 (77.2)		26 (72)		181 (80.1)	
	African American	26 (22.8)		21 (14.2)		11 (13)		36 (20.0)		10 (28)		37 (16.4)	
	Other	4 (3.5)		4 (2.7)		3 (4)		5 (2.8)		0 (0)		8 (3.5)	
Men, n (%)			66 (57.9)		84 (56.8)		45 (55)		105 (58.3)		19 (53)		131 (58.0)
High school education or above, n (%)^b^			44 (38.6)		55 (37.2)		32 (39)		84 (46.7)		20 (56)		86 (38.1)
Potential caregiver not spouse, n (%)^c^			49 (43.0)		104 (70.3)		58 (71)		95 (52.8)		25 (69)		128 (56.6)
Alcohol etiology, n (%)			61 (53.5)		71 (48.0)		39 (48)		93 (51.7)		16 (44)		116 (51.3)
Prior HE^d^, n (%)			66 (57.9)		85 (57.4)		43 (52)		108 (60.0)		25 (69)		125 (55.3)
Lactulose use, n (%)			65 (57.0)		87 (58.8)		45 (55)		107 (59.4)		24 (67)		128 (56.6)
Rifaximin use, n (%)			38 (33.3)		63 (42.6)		34 (41)		67 (37.2)		15 (42)		85 (37.6)
Taking psychoactive medications, n (%)			25 (21.9)		32 (21.6)		16 (20)		41 (22.8)		9 (25)		48 (21.2)
Taking opioids, n (%)			28 (24.6)		43 (29.1)		25 (30)		46 (25.6)		9 (25)		62 (27.4)
MELD^e^ score on admission, mean (SD)^f^			17.9 (7.8)		18.8 (8.9)		18.4 (9.3)		18.4 (8.0)		21.5 (8.4)		17.9 (8.3)
**Reason for admission, n (%)**													
	Infection	37 (32.5)		44 (29.7)		27 (33)		54 (30.0)		14 (39)		67 (29.6)	
	HE^g^	34 (29.8)		48 (32.4)		24 (29)		58 (32.2)		17 (47)		64 (28.3)	
	Renal or metabolic disease	40 (35.1)		57 (38.5)		27 (33)		70 (38.9)		18 (50)		79 (35.0)	
	Gastrointestinal bleeding^g^	22 (19.3)		28 (18.9)		18 (22)		32 (17.8)		3 (8)		46 (20.4)	
	Other^h^	63 (55.3)		81 (54.7)		45 (55)		99 (55.0)		13 (36)		131 (58.0)	

^a^Significant difference for technology demands (*P*=.02).

^b^Significant difference for technology demands (*P*=.046).

^c^Significant difference for caregiver reluctance (*P*<.001) and perceived study burden (*P*=.006).

^d^MELD: Model for End-stage Liver Disease.

^e^HE: hepatic encephalopathy.

^f^Significant difference for technology demands (*P*=.02).

^g^Significant difference for technology demands (*P*=.001).

^h^Significant difference for technology demands (*P*=.04).

**Table 3:** Further reasons for refusal to participate in relation to patient characteristics (N=262).

Characteristic		Transport/logistics		No specific reason	
		Yes (n=26)	No (n=235)	Yes (n=11)	No (n=251)
Age (years), mean (SD)		62.4 (10.3)	59.0 (11.3)	59.9 (10.8)	59.2 (11.4)
**Race, n (%)**					
	Caucasian	19 (73)	188 (80.0)	10 (91)	197 (78.5)
	African American	6 (23)	41 (17.4)	1 (9)	46 (18.3)
	Other	1 (4)	7 (3.0)	0 (0)	8 (3.2)
Men, n (%)		16 (62)	134 (57.0)	6 (55)	144 (57.4)
High school education or above, n (%)		11 (42)	105 (44.7)	6 (55)	110 (43.8)
Potential caregiver not spouse, n (%)		15 (58)	137 (58.3)	5 (45)	148 (59.0)
Alcohol etiology, n (%)		10 (38)	122 (51.9)	7 (64)	125 (49.8)
Prior HE^a^, n (%)		14 (54)	136 (57.9)	6 (55)	145 (57.8)
Lactulose use, n (%)		15 (58)	137 (58.3)	6 (55)	146 (58.2)
Rifaximin use, n (%)		7 (27)	93 (39.6)	5 (45)	96 (38.2)
Taking psychoactive medications, n (%)		4 (15)	53 (22.6)	4 (36)	53 (21.1)
Taking opioids, n (%)		5 (19)	66 (28.1)	4 (36)	67 (26.7)
MELD^b^ score on admission, mean (SD)		16.3 (6.3)	18.6 (8.6)	17.9 (8.7)	18.4 (8.4)
**Reason for admission, n (%)**					
	Infection	7 (27)	74 (31.5)	2 (18)	79 (31.5)
	HE	11 (42)	70 (29.8)	2 (18)	80 (31.9)
	Renal or metabolic disease	9 (35)	88 (37.4)	3 (27)	94 (37.5)
	Gastrointestinal bleeding	5 (19)	44 (18.7)	3 (27)	47 (18.7)
	Other^c^	7 (27)	137 (58.3)	4 (36)	140 (55.8)

^a^MELD: Model for End-stage Liver Disease.

^b^HE: hepatic encephalopathy.

^c^Significant difference for transport/logistics (*P*=.002).

As shown in Table 4, there were some differences detected between the patients across the three sites with respect to demographic characteristics, whereas MELD score and prior hepatic encephalopathy rates were similar. Veterans were older, likely to be men, and more likely to be on rifaximin, whereas the VCU cohort was more likely to be on psychoactive medications and to be admitted for infection or renal/metabolic reasons. Mayo Clinic patients were more likely to be Caucasian and to be admitted for gastrointestinal bleeding. Although there were differences in the reason for admission of patients at each site, differences between sites were ultimately not significant in the multivariable logistic regression analysis.

**Table 4:** Differences in patient characteristics among the three sites.

Characteristics		VCU^a^ (n=191)	VA^b^ (n=56)	Mayo Clinic (n=102)	*P* value
Age (years), mean (SD)		57.6 (10.6)	62.8 (10.8)	59.9 (12.6)	.006
**Race, n (%)**					<.001
	Caucasian	140 (73.3)	34 (61)	97 (95.1)	
	African American	44 (23.0)	17 (30)	1 (0.9)	
	Other	8 (4.2)	5 (9)	4 (3.9)	
Men, n (%)		103 (53.9)	52 (93)	58 (56.9)	<.001
**Comorbidity, n (%)**					.03
	HCV^c^	22 (11.5)	7 (13)	4 (3.9)	
	HCV+alcohol-related	74 (38.7)	19 (34)	50 (49.0)	
	Alcohol-related	16 (8.4)	6 (11)	2 (2.0)	
	NAFLD^d^	40 (20.9)	18 (32)	27 (26.5)	
	Other	39 (20.4)	6 (11)	19 (18.6)	
Prior HE^e^, n (%)		118 (61.8)	37 (66)	52 (51.0)	.11
Lactulose use, n (%)		118 (61.8)	33 (59)	51 (50.0)	.15
Rifaximin use, n (%)		72 (37.7)	32 (57)	37 (36.3)	.02
Taking psychoactive medications, n (%)		51 (26.7)	8 (14)	10 (9.8)	.001
Taking opioids, n (%)		50 (26.2)	9 (16)	23 (22.5)	.26
MELD^f^ score on admission, mean (SD)		19.3 (8.1)	17.4 (7.1)	18.2 (8.5)	.26
**Reason for admission, n (%)**					
	Infection	80 (41.9)	24 (43)	6 (5.9)	<.001
	HE	71 (37.2)	21 (38)	28 (27.5)	.21
	Renal or metabolic disease	98 (51.3)	10 (18)	18 (17.6)	<.001
	Gastrointestinal bleeding	40 (20.9)	7 (13)	31 (30.4)	.02
	Other	87 (45.5)	23 (41)	55 (53.9)	.23

^a^VCU: Virginia Commonwealth University

^b^VA: Rochester Veteran Affairs Medical Center.

^c^HCV: hepatitis C virus.

^d^NAFLD: nonalcholic fatty liver disease.

^e^HE: hepatic encephalopathy.

^f^MELD: Model for End-stage Liver Disease.

## Discussion

Our results demonstrate that agreeing to participate in an intensive health IT regimen aimed at preventing 30-day readmissions for inpatients with cirrhosis and their caregivers may depend in part on the cirrhosis etiology and reasons for inpatient admission. However, the major reasons cited by dyads for not participating appeared to focus on caregivers, study burden, and technological demands rather than on privacy issues. The goal of this multisite trial is to reduce readmissions in cirrhosis, an intractable issue that has medical, psychosocial, and financial consequences [2,8,9]. Our preliminary study demonstrated reductions in hepatic encephalopathy–related readmissions at 30 days when using an intensive health IT intervention that focused on daily communications among patients, family caregivers, and the clinical team [5]. However, given that this previously tested intervention involved several steps in the daily recording of data that could potentially become onerous for the respondents, we included this semistructured interview study within the multisite trial to examine dyads’ perspectives on the use of health IT interventions to better streamline our approach.

Only 25% of the patient-caregiver dyads who were approached in the hospital agreed to be randomly assigned to standard care or to one of the two health IT interventions. This rate is relatively lower than that of individuals who ultimately decided to download another app, EncephalApp, but was similar to the rate of individuals that ultimately used the technology in a recent study of outpatients with cirrhosis [10]. Unlike EncephalApp, which links to the Patient Buddy app, the regimen tested in our multisite trial involves daily entry of data related to medication adherence, weight, and a multipronged approach to orientation that unfolds over 30 days and includes an educational component. This time frame requires a much longer commitment from patient-caregiver dyads along with training from health care providers at the time of discharge. Nevertheless, the intervention includes valuable educational material and offers the potential to continue communication with the treating teams after discharge for most of the clinical issues that underlie readmission, such as hepatic encephalopathy, gastrointestinal bleeding, ascites, and medication management [3,9]. This approach was tested by Bloom et al [11] in a cross-sectional survey where theoretical acceptance of an app that would require similar communications as currently being tested with Patient Buddy was considered to be acceptable to most patients. This finding is in contrast to the results of this study, likely because unlike the theoretical constructs in the prior study, the dyads had to agree to be randomized for a 30-day trial in real life in our study.

The fact that patients admitted with hepatic encephalopathy and gastrointestinal bleeding were more likely to agree to participate in randomization may be related to the patient-caregiver dyad wanting to avoid such occurrences in the future. By contrast, patients with alcohol-related liver disease etiology and opioid use, which often coexist with lower socioeconomic and education status (and potentially unfamiliarity with apps), were less likely to participate [12]. The burden of cirrhosis, and especially hepatic encephalopathy, is shared by patients and their caregivers [13]. Since the most important reasons behind patient-caregiver dyads not agreeing to proceed further were related to caregiver reluctance, perceived burden of the study, and issues involving transport and logistics, the design and components of the health IT intervention may need reconsideration.

The findings of this study are instructive. Although the overall goal of the study team and the multisite trial is to reduce burden for both patients and their families by preventing readmission, the amount of time and effort required to participate in this study may have mitigated the dyads’ desire or willingness to engage in the activities needed to keep up with data entry in the app [14]. The Patient Buddy app being tested in the multisite trial was streamlined from the version tested in our preliminary work based on patient and caregiver responses (specifically items designed to reduce gait issues, fall risk, and sodium content of the diet, and the complexity of the questions asked). However, given the multiple demands on the patient-caregiver dyads’ time during hospitalization and just prior to discharge, and the complexities of the current Patient Buddy app (even with its improvements), it appears that a better balance between gaining the information required and minimizing the burden on patient-caregiver dyads is warranted.

Apart from the barriers mentioned above, reasons for not wanting to be randomized to using a health IT intervention could also be centered around privacy and technological demands. However, of interest, privacy issues were not cited as a reason for the decision not to undergo randomization, which might signify trust in the HIPAA compliance of these apps or the presence of other more pervasive issues.

With respect to refusals on technological grounds, we found that patients admitted with hepatic encephalopathy and those who were older, less educated, and with higher MELD scores were more likely to refuse participation. Similar results have been shown in prior studies in patients with and without cirrhosis, and may represent a lack of individual or family capability to perform the tasks [15]. Given that we anticipated enrolling patient-caregiver dyads from a lower socioeconomic status in the multisite trial, we specifically designed a study protocol that provided smartphones individually to patients and their caregivers. However, it is still possible that unfamiliarity with a smartphone, including the use of apps, remains a barrier to engagement in health IT interventions in patients with liver disease. Although the potential issues with use of one-time apps, particularly in the outpatient setting, may be overcome with teaching, it is possible that sustained use over 30 days for the Patient Buddy app could have been construed as too demanding for older patients with hepatic encephalopathy and more advanced cirrhosis.

Finally, an important area for discussion is that perspectives of the study team and patient-caregiver dyads may have influenced our results. Although some studies focus on specific areas of cirrhosis case management postdischarge, the Patient Buddy app incorporates a more customized approach to cirrhosis and is also more broadly focused. For instance, factors such as hepatic encephalopathy, ascites, renal and metabolic issues, and gastrointestinal bleeding could all potentially influence readmission risk. Therefore, the comprehensive nature of this app could have resulted in the perception that it was more difficult to use compared to other apps focused on a single complication. Nonetheless, a broadly focused and well-rounded app addressing the impact of all complications of cirrhosis is needed to better educate patients and their family members about the disease and ultimately reduce readmission rates.

Our work with the Patient Buddy app and its impact on readmissions is continuing in the multisite trial, but this interim exploration of reasons for why dyads declined randomization in the trial has provided information to further refine our understanding on how to better address barriers to using health IT interventions. This will result in streamlining our approach toward the dyads and focusing on the time spent and potential benefits as we approach them. The rate of participation was lower compared with that reported in some other app-based trials in gastroenterology that focused on patients undergoing colonoscopy and those with inflammatory bowel disease [16,17]. These differences could be due to the inpatient setting, which selects for advanced disease and cognitive demands of the cirrhosis disease process, likely being greater than those involved for other diseases studied. Moreover, we interviewed patients and caregivers together, which makes it difficult to evaluate individual responses, and we did not specifically evaluate socioeconomic factors. We asked questions regarding technological familiarity rather than providing dedicated questionnaires since the dyads were not interested in performing further study-related work apart from a brief interview.

In conclusion, the reasons for patients with cirrhosis and their caregivers declining to participate in a trial using an intensive app that requires 30 days of feedback regardless of demographics and clinical settings were mainly related to caregiver and study demands, but importantly not to privacy concerns. Those admitted with hepatic encephalopathy and gastrointestinal bleeding issues were more likely to agree to participate, whereas those with alcohol etiology and opioid use were less likely to participate in the health IT study. Further research should address the careful balance of patient and caregiver burdens with clinicians’ needs for accurate and timely information that enables good disease management.

## Abbreviations

HIPAA: Health Insurance Portability and Accountability Act
IT: information technology
MELD: Model for End-stage Liver Disease
OR: odds ratio
VA: Veterans Affairs
VCU: Virginia Commonwealth University
